# Modification of the loops in the ligand-binding site turns avidin into a steroid-binding protein

**DOI:** 10.1186/1472-6750-11-64

**Published:** 2011-06-09

**Authors:** Tiina A Riihimäki, Soili Hiltunen, Martina Rangl, Henri R Nordlund, Juha AE Määttä, Andreas Ebner, Peter Hinterdorfer, Markku S Kulomaa, Kristiina Takkinen, Vesa P Hytönen

**Affiliations:** 1 Institute of Biomedical Technology, University of Tampere and Tampere University Hospital, FI-33520 Tampere, Finland; 2 Institute of Biophysics, Johannes Kepler University Linz, 4040 Linz, Austria; 3 VTT Technical Research Centre of Finland, FI-02044 VTT, Finland

**Keywords:** protein engineering, avidin scaffold, phage display, steroid hormone, testosterone

## Abstract

**Background:**

Engineered proteins, with non-immunoglobulin scaffolds, have become an important alternative to antibodies in many biotechnical and therapeutic applications. When compared to antibodies, tailored proteins may provide advantageous properties such as a smaller size or a more stable structure.

**Results:**

Avidin is a widely used protein in biomedicine and biotechnology. To tailor the binding properties of avidin, we have designed a sequence-randomized avidin library with mutagenesis focused at the loop area of the binding site. Selection from the generated library led to the isolation of a steroid-binding avidin mutant (sbAvd-1) showing micromolar affinity towards testosterone (K_d _~ 9 μM). Furthermore, a gene library based on the sbAvd-1 gene was created by randomizing the loop area between *β*-strands 3 and 4. Phage display selection from this library led to the isolation of a steroid-binding protein with significantly decreased biotin binding affinity compared to sbAvd-1. Importantly, differential scanning calorimetry and analytical gel-filtration revealed that the high stability and the tetrameric structure were preserved in these engineered avidins.

**Conclusions:**

The high stability and structural properties of avidin make it an attractive molecule for the engineering of novel receptors. This methodology may allow the use of avidin as a universal scaffold in the development of novel receptors for small molecules.

## Background

Antibodies are the most widely used biomolecules for therapeutic, diagnostic and research applications, because they can be generated against virtually any molecule using protein engineering techniques (for a review see [1]). However, antibodies have certain fundamental disadvantages such as the complex architecture of their antigen-binding site, low stability, and a rather large size [2-5]. Moreover, the production of full-size antibodies is relatively expensive [6]. To overcome these limitations and to improve therapeutic antibodies, antibodies have been extensively engineered [7]. For example the size of the antibody molecule has been reduced by producing single-domain antigen-binding derivatives [8]. In addition to extensive antibody engineering, a versatile repertoire of tailored biomolecules from non-immunoglobulin protein scaffolds have been generated [4,9,10]. Anticalins, derived from the lipocalin fold, are a good example of engineered proteins [11]. The β-barrel structure of lipocalins is thermostable and robust and serves as an excellent scaffold for engineering novel receptors. They have been modified to bind novel ligands, such as fluorescein and digoxigenin, with affinities comparable with antibodies [12].

Chicken avidin (Avd), known for its extremely high affinity towards the water-soluble vitamin H, D-biotin, has been widely used in life science research applications [13]. Aside from biotin, Avd also binds dyes and peptides, which share no significant structural similarity with biotin [14,15]. Avd provides an attractive robust scaffold for the development of novel receptors, and Avd has many advantageous properties such as high chemical and thermal stability, a deep ligand binding site optimized for the binding of small molecules, and an oligomeric nature enabling signal amplification. Moreover, the structure of Avd is well characterized [16,17] and numerous engineered forms of Avd have been described [18]. Engineered Avd forms, in which the two pairs of the binding sites (dual-chain Avd) [19-21] or all four binding sites (single-chain Avd) [22] can be independently manipulated, have been developed.

In the present study Avd was modified to bind steroid hormones. Avd mutant sbAvd-1, which was captured using the phage display selection [23], has a micromolar affinity to testosterone. The steroid-binding protein sbAvd-1 was characterized and further engineered to decrease cross-reactivity towards other molecules, especially towards Avd's natural ligand biotin. The resulting sbAvd-2 mutant with a modified loop between *β*-strands 3 and 4 was found to prefer steroid hormones over biotin in ligand binding.

## Results

### Functional display of Avd protein on the M13 phage

**Figure 1:**
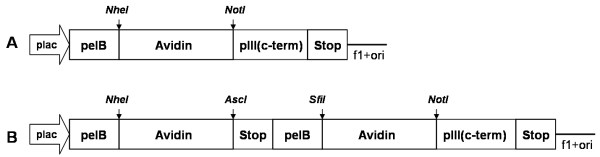
**Schematic presentation of the Avd display expression constructs**. (A) The phagemid constructs for Avd and Avd(N118M) display, in which Avd protein is produced solely as a fusion with pIII. (B) The phagemid constructs for Avd/Avd-pIII and Avd(N118M)/Avd(N118M)-pIII display, in which the *pelB *signal sequence is used for secretion of the Avd-pIII fusions and the free Avd. The cloning sites used are shown by vertical arrows.

Avd was displayed on the surface of the M13 phage as a fusion with the C-terminal region of the minor coat protein pIII. Two different strategies for displaying the Avd scaffold in the active form on the M13 phage were evaluated. In the first display construct, Avd was produced solely as a fusion with pIII (Avd-pIII; Figure 1A), whereas in the Avd/Avd-pIII display construct, free Avd subunits were produced in addition to the pIII fusion (Figure 1B).

**Figure 2:**
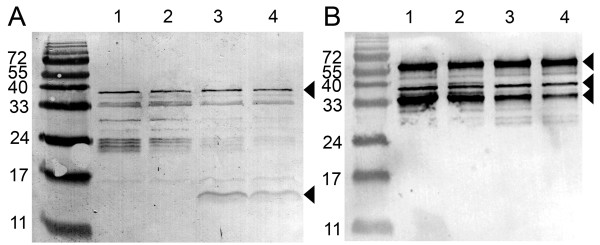
**Immunoblot analysis of Avd phages with anti-Avd and anti-pIII antibodies**. (A) The location of the Avd-pIII fusion protein recognized by anti-Avd is indicated by the upper arrowhead. The theoretical mass of the Avd-pIII fusion protein is 38 kDa. The produced free Avd (~14 kDa) is indicated with the lower arrowhead. (B) From the immunoblot analyzed with anti-pIII antibody the full-length pIII expressed from the helper phage (VCSM13) can be seen migrating at ~60 kDa (the upper arrowhead). The Avd-pIII fusion is indicated by the middle arrowhead. In addition, some proteolytically truncated Avd-pIII forms were detected (~33 kDa; lower arrowhead). Lane 1: Avd-pIII phage; lane 2: Avd(N118M)-pIII phage; lane 3: Avd/Avd-pIII phage; lane 4: Avd(N118M)/Avd(N118M)-pIII phage. The molecular weights of standard proteins are shown as kilodaltons on the left side of each blot.

The size and oligomeric state of the Avd-pIII fusions were analyzed by SDS-PAGE and by western blot. Polyclonal anti-Avd (Figure 2A) indicated expression of both the Avd-pIII fusion (upper arrow, ~38 kDa) and the free Avd (lower arrow, ~15 kDa). The production of free subunits should enhance the functional assembly of the tetrameric Avd scaffold, especially if membrane anchoring of Avd by the pIII fusion partner has a negative effect on the oligomerization of Avd subunits. This strategy mimics the generally used amber-stop codon technique, in which free subunits are produced by the read-through of amber stop codon by the tRNA. We assume that the oligomerization of Avd, secreted by the *pelB *signal sequence, occurs in the periplasmic space of *E. coli*, allowing the display of functional Avd on the phage, as is the case for the display of the antibody Fab fragment, which requires folding of the heavy and light chains for the assembly of a functional antibody molecule [24].

A portion of the Avd-pIII fusion was partially proteolytically cleaved, as can be seen from the blot analyzed with the anti-pIII monoclonal antibody (Figure 2B, ~35 kDa band). Because Avd display constructs designed in this study were based on the monovalent display mode (3 + 3), the intact pIII (migrating at ~58 kDa) expressed from the helper phage was also detected from the blot (Figure 2B, uppermost arrow).

Avd-displaying phages were functional because they bound specifically to the biotin-coated surfaces, and phages were efficiently amplified even after several panning rounds. Moreover, phagemid DNA with the Avd display expression unit, which was confirmed by the restriction enzyme digestion analysis, was stable during the panning rounds (data not shown).

To analyze the functionality of phages, the mixture of amplified Avd and Avd mutant N118M phages were screened by panning against 4-hydroxyazobenzene-2-carboxylic acid (HABA). As determined in our previous study, the biotin binding affinity of the Avd mutant N118M was reduced ~1,000,000-fold (K_d _= 4.2 × 10^-9 ^M) and HABA-affinity was increased ~1.5-fold (K_d _= 5.2 × 10^-6 ^M) compared to wtAvd [25] (Additional file 1). During the panning procedure a clear enrichment of Avd(N118M) phages over wt Avd phages was detected, which was an indication of the high selectivity of the produced phages. After only three rounds of selection phages displaying the Avd mutant N118M outcompeted the wtAvd phage population (Additional file 2).

### Capture of a steroid-binding avidin

**Figure 3:**
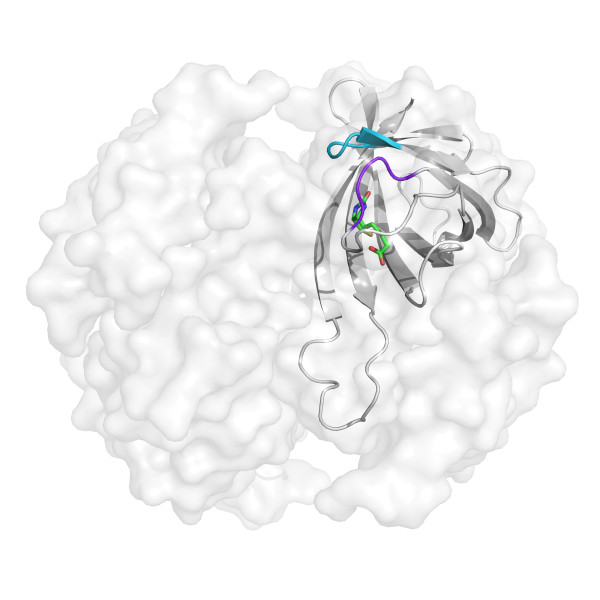
**Three-dimensional structure of wtAvd with the loops chosen for random mutagenesis marked**. One subunit of tetrameric wtAvd with bound biotin (PDB: 2AVI) [16] is shown in the figure. The randomized amino acid residues are N12, D13, L14, G15, and S16 (shown in blue) in the loop between β-strands 1 and 2, and T35, A36, V37, and T38 (shown in purple) in the loop between β-strands 3 and 4.

The loops adjacent to the ligand-binding site were selected for randomization [16]. First we created a library (Avd L1,2 library) in which residues N12, D13, L14, G15, and S16 were randomized. These amino acids form a loop between *β*-strands 1 and 2 (Figure 3). Codon NNN was used for randomization, and therefore, all 20 different amino acids were present, including all stop codons. The Avd L1,2 library was ligated into a phagemid as a sole fusion with the C-terminal portion of pIII. Based on sequencing results and transformation efficiency, the Avd L1,2 library was found to consist of approximately 1 × 10^5 ^individual members. When a stretch of five amino acid residues is completely randomized, the theoretical library size is 3.2 × 10^6^.

In the panning experiments Avd L1,2 loop library phages were introduced onto a testosterone-coated surface. The phage genomes carrying the mutated cDNA were found to be stable during the selection. A clear enrichment of sequences in the randomized loop area was observed, indicating the success of the selection conditions. Interestingly, we detected N12 as being a highly conserved amino acid residue among the enriched pool of proteins. An Avd variant, named sbAvd-1, that carried the sequence N12, R13, M14, N15, H16 was selected for further analysis.

### The specificity of sbAvd-1 can be tuned by additional mutations in the loop between β-strands 3 and 4

To further lower the biotin-binding affinity and to decrease the cross-reactivity of steroid-binding Avd, a library (sbAvd-1 L3,4) was generated in which the loop area between *β*-strands 3 and 4 was randomized (Figure 3). The loop between *β*-strands 3 and 4 is highly important for biotin binding of Avd because this loop 'locks' biotin into the binding site. In this process, three amino acid residues in the loop form direct interactions with biotin [16]. In the library four amino acids (T35, A36, V37 and, T38) were randomized using the NNY codon. The use of this codon covers 14 of the 20 naturally occurring amino acids while eliminating all of the stop codons. The library consisted of approximately 1.4 × 10^6 ^individual members, when calculated from the sequencing results and transformation efficiency, which exceeds the theoretical size of the library calculated based on the possible combinations of amino acid residues (3.8 × 10^4^).

Binders from the sbAvd-1 L3,4 library were selected by phage display panning against a testosterone surface. In every panning round washes were adjusted according to the number of output colonies. The quality of the phage genomes carrying the mutated cDNA was evaluated by DNA sequencing at various stages during selection. A combination of acid and testosterone was used for elution. The selected sbAvd-1 phage clones were evaluated by microplate analysis using BSA-testosterone as a target ligand and utilizing M13-antibody to determine the amount of bound phages. The sbAvd-1 variant that showed the highest binding activity in the microplate assay (data not shown) had the sequence A35, T36, V37, N38. This mutant, named sbAvd-2 was selected for comparative analysis with sbAvd-1.

### Production and purification of Avd mutants

Proteins were produced in soluble form in the *E. coli *strain BL21-AI using N-terminal OmpA bacterial secretion signal from *Bordetella avium *[26]. Proteins were purified by Ni-NTA affinity chromatography that yielded ~2 mg/L pure protein. According to gel-filtration analysis, in solution, both sbAvd-1 (51 kDa) and sbAvd-2 (53 kDa) showed tetrameric state (Additional file 3) similar to that of wtAvd (Avd expressed in *E. coli *53 kDa, chicken Avd 60 kDa [26]). The slight decrease in molecular weight of Avd expressed in bacteria can be explained by the lack of glycosylation.

### Determination of ligand-binding specificity of Avd forms by microplate assay

**Figure 4:**
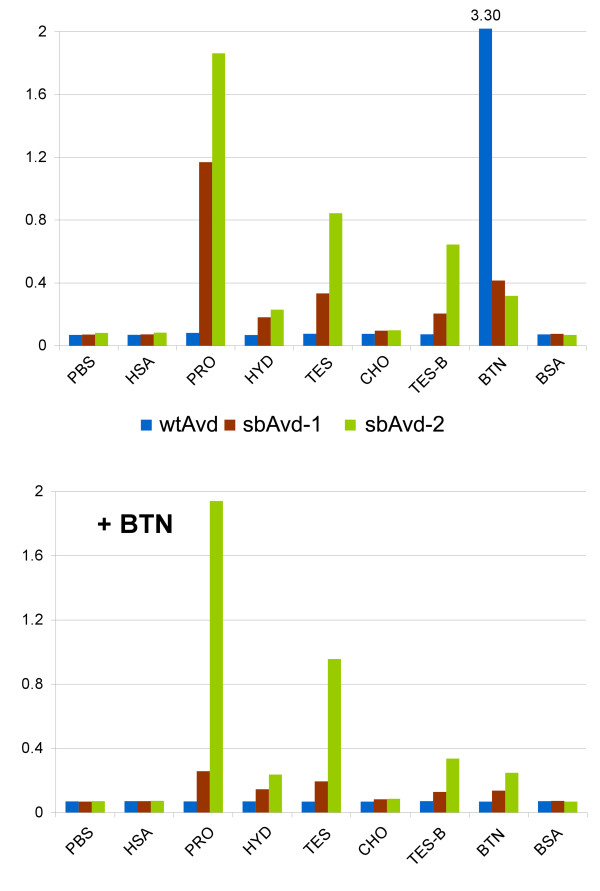
**Determination of ligand-binding specificity of sbAvd-1 and sbAvd-2 proteins by microplate analysis**. The binding of sbAvd-1 and sbAvd-2 to a set of different small ligands was detected using polyclonal anti-avidin antibody as a probe (A) and the effect of free biotin (10 μM) to ligand-binding was analyzed (B). Abbreviations used in the figure: PBS, Phosphate buffered saline; HSA, human serum albumin; PRO, HSA-conjugated progesterone; HYD, HSA-conjugated hydrocortisone; TES, HSA-conjugated testosterone; CHO, HSA-conjugated cholic acid; TES-B, BSA-conjugated testosterone; BTN, BSA-conjugated D-biotin; BSA, bovine serum albumin.

The binding specificity of proteins was analyzed by microplate assay, in which a set of different small molecules were used as a target molecules (Figure 4A). WtAvd was used as a negative control, and we detected no affinity towards the ligands except biotin. Importantly, sbAvd-1 and sbAvd-2 did not bind the proteins used as carriers for small molecules (bovine serum albumin (BSA) and human serum albumin (HSA)). However, these modified Avds showed clear binding to testosterone and progesterone.

The binding to the surface-immobilized ligands was inhibited by pre-incubating the proteins with 10 μM D-biotin (Figure 4B). Free biotin significantly inhibited the binding of sbAvd-1 to steroids, indicating notable affinity towards biotin. However, in the case of sbAvd-2 the binding of testosterone and progesterone was not affected by biotin, showing a clear decrease in biotin-binding affinity.

### Biosensor analyses of steroid-binding Avds

The kinetic constants of testosterone- and biotin-binding to steroid-binding proteins were determined with surface plasmon resonance (SPR) analysis. The purified sbAvd-1 and sbAvd-2 bound to a testosterone-BSA-coated sensor chip with similar affinities (Table 1), whereas wtAvd showed no binding to the testosterone surface. Furthermore, testosterone-binding was inhibited with varying testosterone concentrations (0.75-50 μM, data not shown). In the case of sbAvd-1, the 50% inhibition was achieved between 5-10 μM testosterone, which is consistent with the determined testosterone surface binding affinity. Interestingly, the 50% inhibition was already achieved in 750 nM testosterone concentration in the sbAvd-2 measurements. This result suggests a much higher binding affinity towards free testosterone than that measured towards surface-immobilized testosterone.

**Table 1:** Kinetic parameters and the determined thermostability of sbAvds

Protein	Ligand	SPR			DSC		
		**k**_**a **_(1/Ms)	**k**_**d **_(1/s)	**K**_**D **_(M)	**T**_**m **_(°C)	Δ**T**_**m **_(°C)	**ΔH **×10^4 ^(cal/mol)
wtAvd	-	-	-	-	85.5	-	5.4
wtAvd	Btn	n.d.	n.d.	n.d.	123.2	37.7	12.8
wtAvd	Tes	-	-	no binding	86.2	0.7	5.6
sbAvd-1	-	-	-	-	80.6	-	5.2
sbAvd-1	Btn	4.2 × 10^5^	5.6 × 10^-4^	1.4 × 10^-9^	83.2	2.6	6.4
sbAvd-1	Tes	1.0 × 10^3^	9.5 × 10^-3^	9.0 × 10^-6^	81.5	0.9	5.7
sbAvd-2	-	-	-	-	82.5	-	4.6
sbAvd-2	Btn	1.0 × 10^3^	6.8 × 10^-4^	6.6 × 10^-7^	83.0	0.5	4.2
sbAvd-2	Tes	813	8.5 × 10^-3^	1.1 × 10^-5^	83.1	0.6	4.8

The kinetic parameters determined by SPR analysis. The biotin binding affinity of avidin is too high to be determined by SPR. The transition midpoint of thermal unfolding (T_m_) and the calorimetric heat of unfolding were determined by DSC. Delta T_m _represents the increase of T_m _in the presence of 50 μM ligand.

The biotin binding of the steroid-binding proteins was analyzed by the biotin-coated sensor chip. The comparison of sbAvd-1 and sbAvd-2 revealed that the biotin-binding affinity decreased almost 500-fold due to the modification of the loop between *β*-strands 3 and 4 (Table 1). This result appears to be consistent with the previous study in which mutation of T35A alone decreased the biotin-binding affinity of wtAvd approximately 200-fold [20].

**Figure 5:**
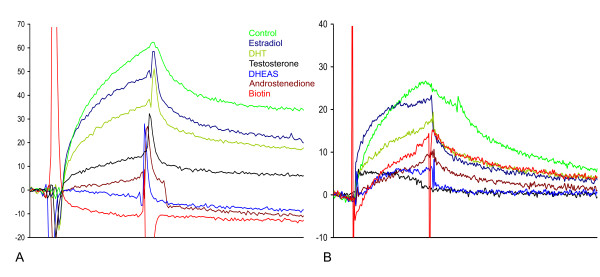
**Inhibition analysis of sbAvd-1 and sbAvd-2 proteins by the SPR method**. The binding of the sbAvd-1 and the sbAvd-2 to a CM5 sensor chip functionalized with testosterone-BSA was measured in the presence of 50 μM inhibitors. (A) The binding of the sbAvd-1 protein was totally inhibited by dehydroepiandrosterone, androstenedione, and biotin. (B) The binding of the sbAvd-2 protein was totally inhibited by dehydroepiandrosterone or testosterone, but not biotin. This result is due to the markedly decreased affinity of the protein towards biotin. Samples: Protein sample, green; protein with estradiol, dark blue; protein with DHT, olive; protein with testosterone, black; protein with DHEAS, blue; protein with androstenedione, brown; protein with biotin, red.

The specificity of the steroid-binding was analyzed by binding competition using the following steroids: testosterone, dehydroepiandrosterone sulfate (DHEAS), androstenedione, estradiol, and dihydrotestosterone (DHT). In addition, the binding to the surface was also competed for by biotin. SbAvd-1 was found to be cross-reactive with androgens highly similar to testosterone (DHEAS and androstenedione), and had noticeable affinity towards biotin (Figure 5A). Interestingly, DHT showed less efficient inhibition compared to testosterone, suggesting lower binding affinity towards this steroid form. The binding of sbAvd-2 to testosterone was found to be most efficiently inhibited by testosterone and DHEAS (Figure 5B), whereas the inhibition caused by biotin was clearly weaker than that in the case of sbAvd-1. This result is consistent with the microplate analysis results and with the binding kinetic constants determined with SPR analysis.

### Interaction analysis of steroid-binding Avds by MRFS

**Figure 6:**
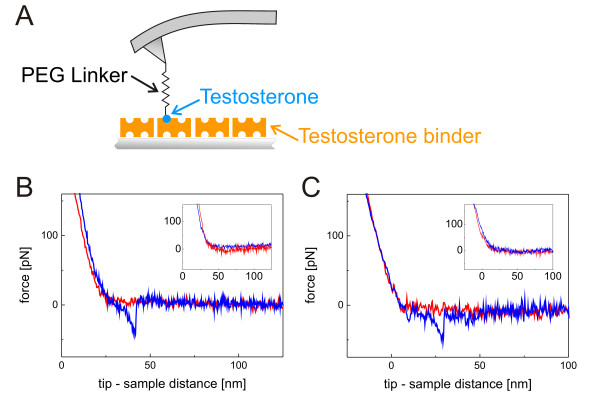
**Analysis of sbAvd protein-testosterone interaction by MRFS method **(A) A schematic representation of the experimental assembly used in the analyses. Testosterone was tethered to the AFM tip using a flexible PEG crosslinker. The steroid-binders sbAvd-1 and sbAvd-2 were covalently bound to the mica surface via a short homobifunctional spacer. (B) The force-distance cycle of the sbAvd-1 - testosterone interaction showing an unbinding event. The typical non-linear shape of the event results from the elastic properties of the PEG linker. (C) The force-distance cycle showing a sbAvd-2 - testosterone bond dissociation. Insets in (B) and (C) represent force-distance cycles in which the protein-testosterone interaction is inhibited with free testosterone.

Molecular recognition force spectroscopy (MRFS) [27] was used to study the interaction between sbAvds and testosterone on a single molecule level. An atomic force microscopy (AFM) tip was functionalized with a single testosterone molecule [28] and repeatedly approached and retracted from the sbAvd-1- or sbAvd-2-coated surface (Figure 6A). The binding forces were measured in force-distance cycles, whereby the deflection (force) of the cantilever was recorded as a function of the tip-sample distance. For evaluation, 1,000 force-distance cycles were recorded, and probability density functions (pdf) were generated from the detected interaction forces [29]. The tip-tethered testosterone was found to form a complex with the sbAvds; the retraction led to a downward bending of the cantilever until a particular force was reached resulting in the rupture of the bond between testosterone and sbAvd (Figure 6B and 6C). The most probable unbinding force [29] was found to be similar for both of the testosterone-sbAvd complexes: 40 pN at a constant pulling velocity of 600 nm/s. From 1,000 recorded force-distance cycles, sbAvd-1 showed 183 detected interactions and sbAvd-2 showed 215 events.

To prove the specificity of the measured interactions, control experiments were performed. For these experiments, free testosterone was injected into the measuring solution to preoccupy the binding sites of the steroid-binding protein immobilized on the surface. The retraction curve identical to the approaching curve was detected (insets of Figure 6B and 6C), which indicated the total inhibition of the binding by free testosterone. Additionally, when the binding sites were preoccupied with free testosterone, the number of binding events dropped down to 67 from 183 detected interactions in the case of sbAvd-1. In case of sbAvd-2 the number of interactions dropped from of 215 to 21 after addition of free testosterone.

### Differential scanning calorimetry reveals the high stability of the engineered proteins

The high thermal stability of wtAvd (temperature transition midpoint (T_m_) = 85.5°C) was preserved in the steroid-binding mutants; these proteins showed similar T_m _values compared to wtAvd (sbAvd-1 T_m _value of 80.6°C and sbAvd-2 T_m _value of 82.5°C) (Table 1, Additional file 4). Ligand binding often stabilizes a protein and raises the T_m _value. As expected, the addition of biotin stabilized wtAvd effectively, increasing the T_m _value almost 40°C. This T_m _indicates a very high binding affinity.

The effect of biotin-binding on the protein stability of steroid-binding proteins was much smaller than in the case of wtAvd, thus showing decreased biotin affinity. In fact, sbAvd-2 showed a negligible increase in T_m _in the presence of biotin (ΔT_m _= 0.5°C). The presence of testosterone only slightly increased the T_m _values of sbAvds (ΔT_m _= 0.6-0.9°C). Actually, a similarly small increase was detected in the ΔT_m _value of wtAvd when testosterone was added. However, because the testosterone-binding of wtAvd was not detected in other analyses (SPR and microplate analysis) the mechanism of stabilization remains unclear.

## Discussion

In the present study, Avd proteins were displayed on the M13 phage in a monovalent form fused with the C-terminal region (aa residues 198-406) of the coat protein pIII. The crucial requirement for the functional display on phage is successful expression of the protein in *E. coli*. As has been earlier reported Avd protein can be efficiently expressed in a soluble form in *E. coli *[26]. The close relative of Avd, streptavidin, has been displayed on bacterial phages as a pVIII fusion [30,31]. However, to our best knowledge, chicken Avd has not been previously displayed on phages for screening purposes.

To evaluate the different modes for Avd display, we generated constructs expressing pIII fused to Avd and constructs that expressed free Avd subunits in addition to the pIII fusions. Based on the expression analysis, binding properties and selection experiments, the construct that expressed both fusion and free Avd showed an enhanced assembly of functional, tetrameric Avd on the phage compared to the Avd-pIII fusion alone (Additional file 2). However, the construct that expressed pIII-fused Avd was chosen for use in the selection system for the generation of gene libraries because the use of only one Avd gene in the protein display would ensure that the selected proteins could be assembled to homotetramers.

The potential of the developed platform for screening novel Avd-based receptors was investigated. Five residues in the loop between *β*-strands 1 and 2 of Avd were randomized to generate a population of diverse Avd genes. The gene population was screened for novel binding properties using the phage display method [23]. We observed the enrichment of Avd phages binding to testosterone-coupled BSA. The Avd variant sbAvd-1, which carried the sequence N12, R13, M14, N15, H16, was further analyzed and found to bind free testosterone with an affinity similar to BSA-conjugated testosterone. Moreover, we detected that the biotin-binding affinity of sbAvd-1 was not completely diminished; it was still high enough to inhibit sbAvd-1 from binding to testosterone-BSA (Table 1, Figures 4 and 5). This finding confirmed that testosterone occupies the same binding site in Avd as does biotin. Based on the cross-creativity measurements, sbAvd-1 also binds other steroid hormones, such as progesterone and DHEAS (Figures 4 and 5).

SbAvd-1 was further engineered to decrease biotin binding and to improve steroid specificity. From the phage display selections, the sbAvd-1-derived protein, named sbAvd-2, with a mutated loop between β-strands 3 and 4 was captured. This mutant showed similar or slightly decreased binding affinity towards immobilized testosterone, whereas the biotin-binding affinity was clearly decreased (Table 1, Figures 4 and 5). We also noticed that sbAvd-2 bound more tightly to free testosterone than did sbAvd-1 (Figure 5). Significantly, sbAvd-2 preferred steroids as a ligand over biotin; this finding is important when considering the applications for biofluids, in which biotin is often present in relatively high concentrations.

This study and our preliminary results from experiments with a number of different target ligands (Hiltunen S, Riihimäki TA *et al*., unpublished data) suggest that Avd-based receptors for various different small molecules can be tailored. These Avd-based receptors may be valuable tools for diagnostic use in the future.

## Conclusions

The current study provides a promising platform for the selection of tailored ligand-binders evolved from the Avd scaffold in the monovalent pIII protein display. The Avd scaffold has characteristics that are beneficial in protein engineering, such as high thermal and chemical stability, simple folding and an optimal structure for small ligands. Furthermore, Avd can be modified rather freely without major change in the fold [18]. Novel Avds that can simultaneously bind multiple ligands could become next-generation molecular tools for clinical and diagnostic applications [22,32]. Importantly, novel Avd-based receptors could be used in applications that require harsh conditions.

## Methods

### Construction of phagemid vectors

All of the basic recombinant DNA methods were performed essentially as previously described [33]. Appropriate restriction sites were added to the cDNA of the Avd core sequence [34] by PCR using the primers Avd_NheI_5' and Avd_NotI_3' (Additional file 5). PCR products were first subcloned into the pCR^®^2.1-TOPO plasmid by TOPO TA-cloning (Invitrogen) and the plasmids were transformed into *E. coli *TOP10 cells. Plasmids were isolated from colonies that contained the inserts based on the blue-white screening. Avd fragments were cut out from the pCR^®^2.1-TOPO-plasmid using the NheI and NotI restriction enzymes and ligated into the phagemid vector (pBluescript SK+ derived phagemid, Research Center of Finland, Biotechnology, Espoo, Finland). The cDNA of the Avd was cloned into the phagemid vector as an N-terminal fusion to the C-terminal domain (amino acids 198-406) of the minor phage coat protein III. In the Avd/Avd-pIII constructs, the coding sequence of the free Avd was subcloned using the primers Avd_NheI_5' and Avd_AscI_stop_3' (Additional file 5). The Avd-pIII expression cassette was generated by subcloning a fragment amplified from the primers Avd_SfiI_5' and Avd_NotI_3' (Additional file 5). The nucleotide sequences of the Avd constructs were verified by sequencing on an ABI PRISM 3100 Genetic Analyzer (Applied Biosystems) according to the protocols recommended by the manufacturer (ABI PRISM BigDye Terminator Cycle Sequencing Kit v.1.1, Applied Biosystems).

### Amplification of phage particles

All of the basic phage display methods were performed essentially as previously described [35]. Phagemid vectors with the Avd insert were transformed into chemically competent *E. coli *XL1-Blue cells (Stratagene, La Jolla, CA) with the heat shock method. Phage stocks of the different Avd display constructs were made from individual colonies picked from the transformation plates into super broth (SB) medium supplemented with the appropriate antibiotics and glucose. The bacterial cultures were infected with the helper phage (10^12 ^pfu/ml) VCS-M13 (Stratagene, LaJolla, CA) for amplification of Avd phages. Phages were PEG precipitated and analyzed by SDS-PAGE and western blotting following immunostaining with the polyclonal rabbit α-avd IgG (University of Oulu, 1:5000) and the monoclonal mouse anti-pIII IgG (Biosite, Sweden, 1:2000) antibodies.

### Functionality test of Avd-displaying phages

The functionality of the Avd phages was tested by panning the phages on surfaces coated with BSA conjugated to HABA (HABA-BSA) [36]. As a positive control for selection, the Avd mutant N118M was also displayed on the phages. Panning was performed essentially as previously described [35]. For elution, vigorous shaking in 100 mM hydrochloric acid containing 10 μM D-biotin (Biochemica, Fluka, 14400) was performed. In total, three selection rounds were performed. After each round the integrity of the Avd expression units was analyzed by restriction enzyme digestions of the phagemid DNA samples. Importantly, both single and double Avd and Avd(N118M) construct phages were competed against each other. Equivalent amounts of phages displaying Avd or Avd(N118M) mutant were mixed and biopanned. The ratio between constructs was determined with DNA sequencing.

### Construction of Avd L1,2 library

To construct an Avd DNA library, amino acids N12, D13, L14, G15, and S16 in the loop between beta strands 1 and 2 (L1,2) were randomized. The libraries were constructed essentially as previously described [35]. A nucleic acid fragment of 105 base pairs was amplified with the primers Avd_NheI_5' and Loop 1-2 _R1_3' (Additional file 5) using wt Avd cDNA as template. Parallel to this fragment, a nucleic acid fragment with 357 base pairs was PCR-amplified with the primers Loop 1-2_R2_5' and Avd_NotI_3' (Additional file 5) using wt Avd as a template. The PCR strategy is presented in Additional file 6. The desired amplification products were separated by agarose gel electrophoresis and isolated from the gel using the Nucleo Spin Extract II kit (Macherey-Nagel) according to the manufacturer's instructions. PCR products were combined in the second amplification step in the presence of the PCR primers Avd_NheI_5' and Avd_NotI_3' (Additional file 5). This amplification resulted in a DNA fragment of 462 base pairs. The fragment was isolated from the gel and cut with the restriction enzymes *NheI *and *NotI *(Fermentas) followed by purification using the Nucleo Spin Extract II kit (Macherey-Nagel). Fragments were ligated into the phagemid vector and the resulting ligation product was transformed into electrocompetent cells of the *E. coli *strain XL1-Blue (Stratagene) by electroporation. Transfected bacteria were infected with the VCS-M13 helper phage (Stratagene) and phages were harvested from the culture and purified with PEG precipitation. The amount of phage particles was determined by titration.

### Selecting steroid-binders from the Avd L1,2 library

The Avd L1,2 library was panned against the steroid hormone testosterone. As a negative control, wtAvd-displaying phages were also panned against testosterone. NUNC immunosorp plates were coated with a testosterone-BSA conjugate (Sigma, T-3392, 1 μg). Phages were preincubated in the BSA-coated wells to prevent non-specific binding. Three to four selection cycles were conducted, and the stringency of washing conditions was increased every panning round to decrease nonspecific binding. In the first panning round wells were washed three times with phosphate buffered saline containing 0.05% Tween (PBS-Tween) and five times with phosphate buffered saline (PBS). Phages were eluted by vigorous shaking for 10 minutes in 100 mM hydrochloric acid containing 10 μM D-biotin (Biochemica, Fluka, 14400). Biotin was used for elution because it was probable that after randomization of the loop L1,2 the resulting Avd would still have a rather high affinity towards biotin. In the final panning round 10 μM testosterone (Steraloids Inc., USA) was used in addition to 100 mM hydrochloric acid for elution. The eluted phage solutions were neutralized with 2 M Tris. Eluted phages were used to infect *E. coli *XL1-Blue cells and aliquots of the infected bacteria were plated to quantify the amount of eluted phages. Phages were amplified and purified as described earlier. Precipitated phages were then used for the next round of selection. After every panning round, results were verified by sequencing (20 sequences), and the number of phage particles was determined by phage titration.

### Generation and screening of the SbAvd-1 L3,4 library

An SbAvd-1-derived DNA library was constructed and ligated into the phagemid as described above. In the library amino acids T35, A36, V37, and T38 in the loop between beta strands 3 and 4 (L3,4) were randomized; thus, in the construction of the library the primers Avd_NheI_5' and 3_4R_1_3' and primers 3_4R_2_5' and Avd_NotI_3' (Additional file 5) were used in the PCR. SbAvd-1 cDNA was used as a template. Ligated phagemid was transformed into electrocompetent cells of *E. coli *strain XL1-Blue (Stratagene) by electroporation. Transfected bacteria were infected with the VCS-M13 helper phage (Stratagene) and phages were harvested from the culture and purified with PEG precipitation. The amount of phage particles was determined by titration.

The sbAvd-1 L3,4 library was biopanned against the steroid hormone testosterone similarly as described above, with some exceptions. NUNC immunosorp plates were coated with a testosterone-BSA conjugate (Sigma, T-3392, 200 ng). Phages were preincubated in BSA-coated wells before the panning procedure. Four selection cycles were performed. Phages were eluted by vigorous shaking for 10 minutes in 100 mM hydrochloric acid containing 35 μM testosterone (Steraloid Inc., USA). After each panning round, results were verified by sequencing (20 sequences), and the number of phage particles was determined by phage titration. Additionally, every panning round was screened for binders by anti-M13 as previously described [37]. For the ELISA, (NUNC) immunosorp plates were coated with a testosterone-BSA conjugate (Sigma, T-3392, 1 μg), wells were blocked with 5% milk solution, and detection was performed with Anti-M13/HRP (GE Healthcare) and read with a microplate reader (Bio-Rad 680 XR).

### Production and purification of Avd mutants

For biochemical analyses, the proteins were produced in *E. coli *strain BL21-AI (Invitrogen) using expression vector pET101/D (Invitrogen) [26]. After sonication (Sonics & Materials Vibra Cell™) and DNaseI (New England Bio Labs) treatment of *E. coli *cells, the purification of the proteins was conducted using Ni-NTA affinity chromatography according to the instructions of the manufacturer (QIAGEN).

The oligomeric state of the proteins was assayed with fast protein liquid chromatography (FPLC) gel-filtration using an ÄKTApurifier™ HPLC equipped with a Superdex 200 10/300 GL column (Tricorn, Amersham Biosciences, GE Healthcare). The column was calibrated using the gel-filtration mixture (thyroglobulin, γ-globulin, ovalbumin, myoglobin, and vitamin B12; Bio-Rad Laboratories) as a molecular-mass standard. Sodium phosphate buffer (20 mM, pH 7.4) with 1M NaCl and, 20 mM imidazole was used as the liquid phase. Protein samples of 90-193 μg in a volume of 500 μl were used in the analysis.

### DSC measurements of wtAvd and steroid-binding Avds

Proteins (0.225 mg/ml) were analyzed in sodium phosphate buffer (20 mM, pH 7.4), containing 20 mM imidazole and 1 M NaCl. D-biotin (Biochemica, Fluka, 14400) and testosterone (Steraloids Inc., USA) were diluted with the measurement buffer to a final concentration of 50 μM. All solutions were degassed prior to measurements to avoid air bubbles. An automated capillary VP-DSC instrument (GE Healtcare, MicroCal, Northampton, USA) was used to measure the stability of the proteins with or without ligands. During the measurement, protein samples were heated from 20°C to 130°C at a scanning rate of 120°C/h. Feedback mode was set to low and the filler period was 8 s. Temperature transition midpoints (T_m_) were recorded from the highest peaks and the calorimetric heat changes (ΔH) were calculated using the MicroCal Origin 7 software (GE Healtcare, MicroCal, Northampton, USA).

### Determination of ligand-binding specificity of Avd forms by microplate assay

MaxiSorp F96 microplate wells (NUNC) were coated with 500 ng of conjugated ligand (HSA-conjugated progesterone, hydrocortisone, testosterone, and cholic acid (Technical Research Center of Finland, Espoo, Finland); or BSA-conjugated testosterone (A6958-000, Steraloids Inc., USA), and biotin (Jenni Leppiniemi, University of Tampere, Finland)), and with 500 ng of the carrier proteins HSA and BSA (A7906, Sigma) in 100 μl of PBS for 2 hours 37°C. Carrier proteins were used as negative controls. Plates were washed three times with PBS-Tween, blocked with 0.5% BSA-PBS for 30 min, and then washed again. Proteins (0.9 μg/ml) in sodium phosphate buffer (20 mM, pH 7.4) with 1M NaCl and 20 mM imidazole, were added to the wells and incubated for 1 h. A portion of wtAvd (Belovo, Bastogne, Belgium), sbAvd-1 and sbAvd-2 was preincubated with 10 μM D-biotin (Biochemica, Fluka, 14400). Bound Avd was detected by rabbit α-avd IgG (University of Oulu) and with alkaline phosphatase conjugated goat anti-rabbit IgG (A3937, Sigma). After adding the phosphatase substrate solution (1 mg/ml pNPP (S0942, Sigma) in 1 M diethanolamine pH 9.8, with 0.5 mM MgCl_2_), the plates were read after 15 minutes at A405 with a microplate reader (Bio-Rad 680 XR).

### Biosensor analyses of steroid-binding Avds

A BIAcore X optical biosensor (Biacore, Uppsala, Sweden) was used for the analysis of binding kinetics. Testosterone-BSA was coupled to the carboxymethylated dextran layer of a sensor chip using standard amine coupling chemistry (1000 RU, 40 μl/min flow rate). Samples of sbAvd-1, and sbAvd-2 were diluted in 50 mM sodium phosphate containing 1 M NaCl, and the same buffer was used in the measurements. The binding of the sbAvd-1 and sbAvd-2 samples on testosterone-BSA coated chips was measured and the kinetic constants were determined from the measurements performed with different protein concentrations using the BiaEvaluation software according to the manufacturer's instructions. WtAvd was used as a negative control.

The binding of steroid-binding proteins to the testosterone-BSA surface was competed with free steroid hormone molecules (testosterone, DHEAS, androstenedione, estradiol, and DHT (Steraloids Inc., USA)) and free biotin (Biochemica, Fluka, 14400) to evaluate the specificity of binding. Additionally, steroid-binding was more closely detected by measuring the binding in the presence of varying concentrations (0.75 μM-50 μM) of inhibiting testosterone.

For the determination of biotin-binding kinetics, a sensor chip was prepared as follows: diaminoethylene was first attached to the surface using a mixture containing 0.2 M 1-ethyl-3-(3-dimethylaminopropyl) carbodiimide hydrochloride (EDC) and 0.05 M N-hydroxysuccinimide (NHS) in water. Second, to introduce amino groups to the surface, 1 M ethylenediamine (Fluka 03550) in water was applied. Finally, 5 mM biotin N-succinimidyl ester (Biochemica, Fluka, 14405) in 50% DMSO was injected on the surface (40 μl/min flow rate, ~130 RU; please note that the determination of the bound mass is not very accurate in case of small molecules because the immobilization can change the physicochemical properties of the surface).

### Molecular Recognition Force Spectroscopy experiments

Molecular recognition force spectroscopy (MRFS) was used to study the interaction of the produced proteins with testosterone and biotin. Steroid-binding proteins were covalently bound to modified mica sheets via lysines as previously described [38]. Testosterone was coupled to the AFM tip via the heterobifunctional Fmoc-PEG-NHS crosslinker as previously described [28], resulting in a covalent attachment of testosterone via a flexible spacer (Figure 6A).

All MRFS experiments were performed on a Pico SPM I (Agilent Technologies, Santa Clara, CA). All modified cantilevers used had nominal spring constants between 10-30 pN/nm (Veeco Instruments, Santa Barbara, CA). The effective spring constants of the cantilevers were determined by the thermal noise method [39,40]. Force-distance cycles were completed using a z-range of 200 and 300 nm. Sweep durations were adjusted between 0.25 and 4 s. During one data set of 1000 force-distance curves, the lateral tip position was changed (a few hundred nm) about every 100 curves to ensure that the binding events were statistically reasonable. The specificity of the binding was proved by adding free testosterone (200 nM) into the measuring solution and incubating for approximately 1 h to block the ligand-binding sites of the proteins.

## Abbreviations

AFM: atomic force microscopy; BSA: bovine serum albumin: BTN: D-biotin; DHEAS: dehydroepiandrosterone sulphate; DHT: dihydrotestosterone; DMSO: dimethyl sulfoxide; EDC: 1-ethyl-3-(3-dimethylaminopropyl) carbodiimide hydrochloride; HABA: 4'-hydroxyazobenzene-2-carboxylic acid; HSA: human serum albumin; MRFS: molecular recognition force spectroscopy; NHS: N-hydroxysuccinimide; PBS: phosphate buffered saline; PEG: polyethylene glycol; sbAvd: steroid-binding avidin; SB: super broth; SDS-PAGE: sodium-dodecyl sulfate polyacrylamide gel electrophoresis; wtAvd: wild type avidin.

## Authors' contributions

TAR conceived the study, constructed the Avd libraries, performed most of the experiments, and contributed to the writing of the manuscript; SH participated in the construction of the Avd libraries and in the experimental work and contributed to the writing of the manuscript; JAEM participated in the experimental work and contributed to the writing of the manuscript; MR, AE and PH performed MRFM measurements and contributed to the writing of the manuscript; HRN, MSK and KT conceived the study and supervised the work; VPH conceived and designed the study, supervised the work and contributed to the writing of the manuscript. All authors read and approved the final manuscript.

## Supplementary Material

Additional file 1 **Structural comparison of Avd-BTN and Avd-HABA complexes**. 3D-structures of Avd complexed with biotin (A) and HABA (B). These two ligands have different hydrogen bonding interactions with the amino acids at the binding site. (A) Hydrogen bonds formed between the ureido ring of biotin and Avd are shown here as dashed green lines. (X-ray crystallographic structure (PDB 2AVI)) [41]. (B) A significant difference between biotin and HABA binding to Avd is seen in the interaction of the ligand with asparagine 118. Although other Avd key residues interacting with the BTN ureido ring also interact with HABA, there is no hydrogen bonding partner for N118 in HABA. Again, hydrogen bonds formed between the carboxyl group of HABA and Avd are shown as dashed green lines. The loop between *β*-strands 3 and 4 was not resolved in the avd-HABA -complex (coordinates kindly provided by Prof. Oded Livnah). The figure was made with the VMD program [42]. Click here for file

Additional file 2 **Sequencing results and input from the control selections**. The percentage of wt Avd and Avd(N118M) mutant sequences after sequencing analysis from the different rounds of HABA selection. The amount of input phages is shown as colony forming units (cfu) per milliliter of culture. Click here for file

Additional file 3 **Gel-filtration chromatograms of sbAvd-1 and sbAvd-2 proteins**. The chromatograms of steroid-binding proteins determined at wavelength of 280 nm by gel-filtration. Besides the main peak, there is also a small peak of oligomeric form of the protein in the chromatogram of sbAvd-1 protein (gray curve). It is a typically observation also in the case of wtAvd [26]. The chromatogram of sbAvd-2 protein is shown with black curve. The tetrameric form of the protein is dominant in both samples. Click here for file

Additional file 4 **The effect of ligand-binding to the stability of wtAvd, sbAvd-1 and sbAvd-2 proteins**. DSC thermograms were obtained from the protein sample (0.225 mg/ml) by scanning temperature range of 20°C to 130°C with heating rate of 120°C/min. The analysis was conducted in the absence and presence of ligands (50 μM). Click here for file

Additional file 5 **The cloning primers used in the study**. The restriction enzyme cleavage sites are indicated in *italics*. Click here for file

Additional file 6 **A schematic presentation of the construction strategy of the Avd L1,2 library**. for 1-2 loop library a nucleic acid fragment of 105 base pairs was amplified (Step 1, A) with the primers Avd_NheI_5' and Loop 1-2 _R1_3' (schematically referred in the figure as Avd_mutant_3') using wtAvd cDNA as a template. Parallel to this, a nucleic acid fragment with 357 base pairs, was PCR-amplified (Step 1, B) with the primers Loop 1-2_R2_5' (schematically referred in the figure as Avd_combine_5') and Avd_NotI_3', also using wtAvd as a template. Amplified fragments were combined in a second amplification step in the presence of PCR primers Avd_NheI_5' and Avd_NotI_3', wherein a DNA fragment of 462 base pairs was obtained. Click here for file
