# Supplementary figures and images for: Modification of the loops in the ligand-binding site turns avidin into a steroid-binding protein
Source: BMC Biotechnol. 2011 Jun 9;11:64. doi: 10.1186/1472-6750-11-64 (PMC3201017; doi:10.1186/1472-6750-11-64)

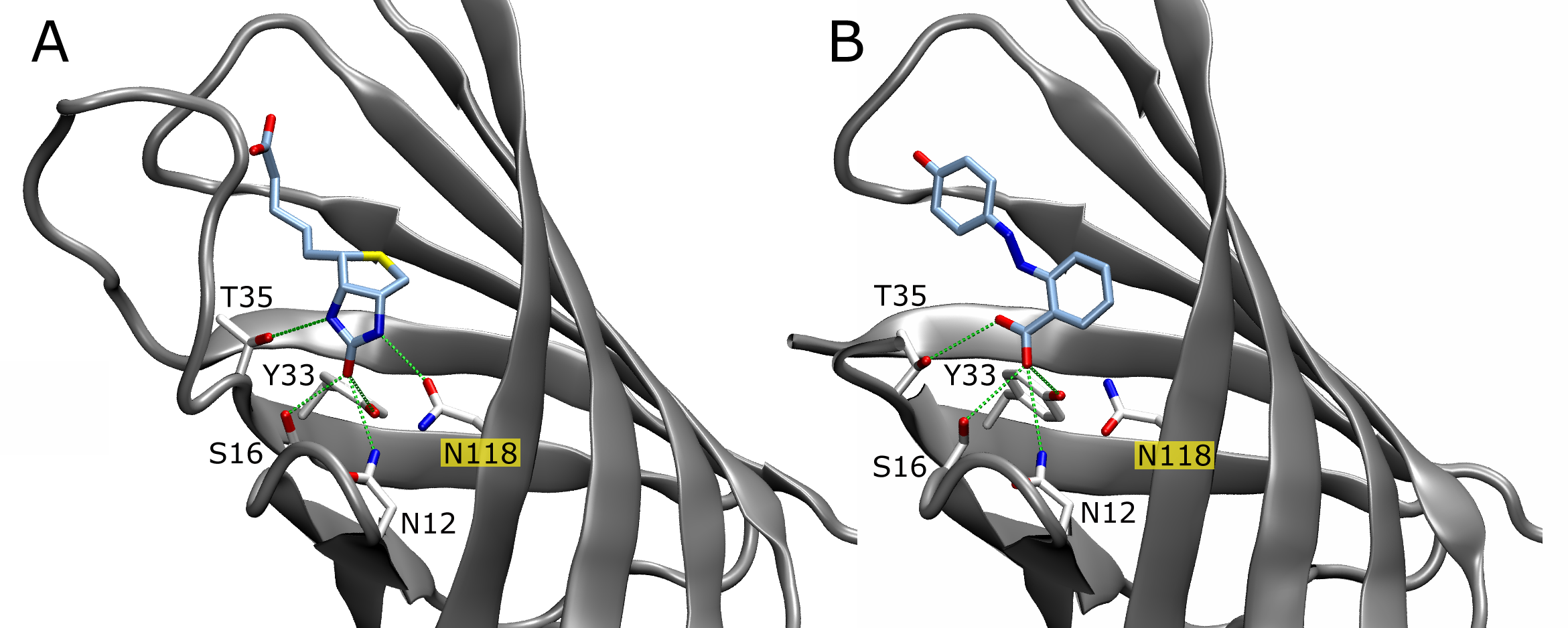

Supplement: Additional file 1 — Structural comparison of Avd-BTN and Avd-HABA complexes. 3D-structures of Avd complexed with biotin (A) and HABA (B). These two ligands have different hydrogen bonding interactions with the amino acids at the binding site. (A) Hydrogen bonds formed between the ureido ring of biotin and Avd are shown here as dashed green lines. (X-ray crystallographic structure (PDB 2AVI)) [41]. (B) A significant difference between biotin and HABA binding to Avd is seen in the interaction of the ligand with asparagine 118. Although other Avd key residues interacting with the BTN ureido ring also interact with HABA, there is no hydrogen bonding partner for N118 in HABA. Again, hydrogen bonds formed between the carboxyl group of HABA and Avd are shown as dashed green lines. The loop between β-strands 3 and 4 was not resolved in the avd-HABA -complex (coordinates kindly provided by Prof. Oded Livnah). The figure was made with the VMD program [42]. [file 1472-6750-11-64-S1.PNG]

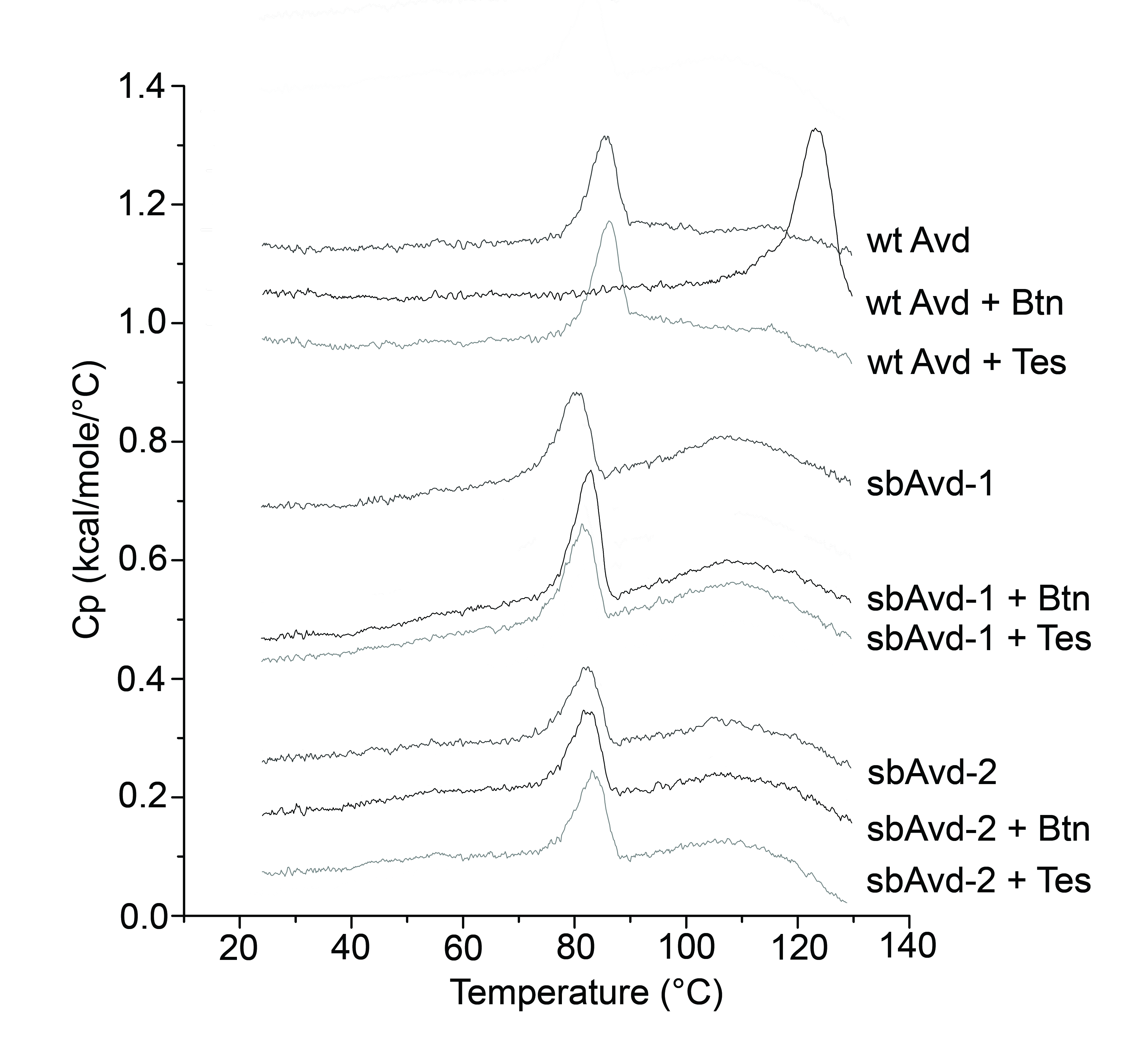

Supplement: Additional file 4 — The effect of ligand-binding to the stability of wtAvd, sbAvd-1 and sbAvd-2 proteins. DSC thermograms were obtained from the protein sample (0.225 mg/ml) by scanning temperature range of 20°C to 130°C with heating rate of 120°C/min. The analysis was conducted in the absence and presence of ligands (50 μM). [file 1472-6750-11-64-S4.TIFF]

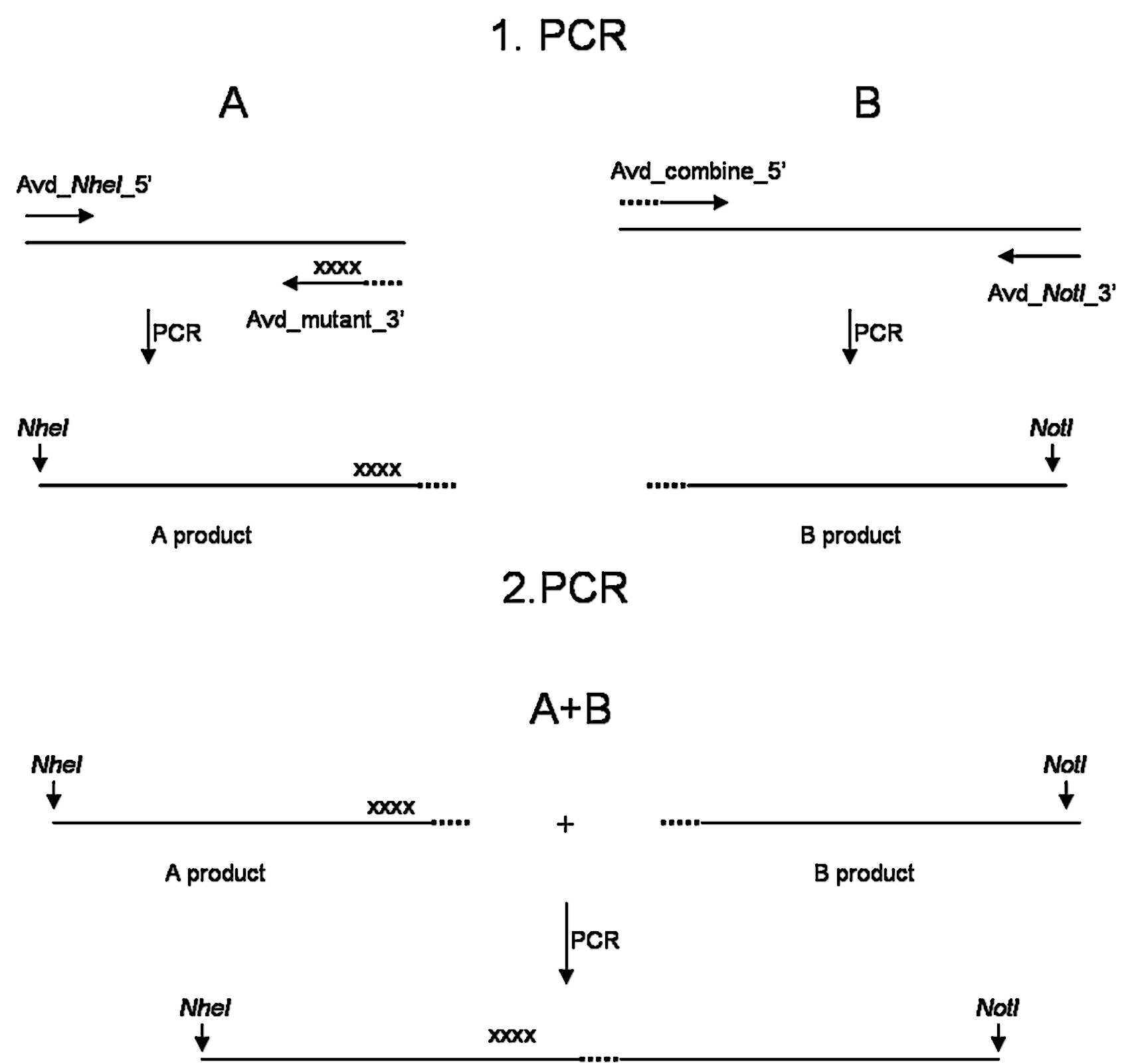

Supplement: Additional file 6 — A schematic presentation of the construction strategy of the Avd L1,2 library. for 1-2 loop library a nucleic acid fragment of 105 base pairs was amplified (Step 1, A) with the primers Avd_NheI_5' and Loop 1-2 _R1_3' (schematically referred in the figure as Avd_mutant_3') using wtAvd cDNA as a template. Parallel to this, a nucleic acid fragment with 357 base pairs, was PCR-amplified (Step 1, B) with the primers Loop 1-2_R2_5' (schematically referred in the figure as Avd_combine_5') and Avd_NotI_3', also using wtAvd as a template. Amplified fragments were combined in a second amplification step in the presence of PCR primers Avd_NheI_5' and Avd_NotI_3', wherein a DNA fragment of 462 base pairs was obtained. [file 1472-6750-11-64-S6.PNG]
