# Supplementary material for: Modification of the loops in the ligand-binding site turns avidin into a steroid-binding protein
Source: BMC Biotechnol. 2011 Jun 9;11:64. doi: 10.1186/1472-6750-11-64 (PMC3201017; doi:10.1186/1472-6750-11-64)
Supplement: Additional file 2 — Sequencing results and input from the control selections. The percentage of wt Avd and Avd(N118M) mutant sequences after sequencing analysis from the different rounds of HABA selection. The amount of input phages is shown as colony forming units (cfu) per milliliter of culture. [file 1472-6750-11-64-S2.DOC]

| Selection round | Input  cfu/ml | **Avd-pIII /**  **Avd(N118M)-pIII**  Seq (%) | Input  cfu/ml | **Avd/Avd-pIII /**  **Avd(N118M)/Avd(N118M)-pIII**  Seq (%) |
| --- | --- | --- | --- | --- |
| 1 | 3000 | 25/75 | 18000 | 33/67 |
| 2 | 400 | 50/50 | 12000 | 25/75 |
| 3 | 3000 | 0/100 | 14000 | 0/100 |
