# Supplementary material for: Modification of the loops in the ligand-binding site turns avidin into a steroid-binding protein
Source: BMC Biotechnol. 2011 Jun 9;11:64. doi: 10.1186/1472-6750-11-64 (PMC3201017; doi:10.1186/1472-6750-11-64)
Supplement: Additional file 5 — The cloning primers used in the study. The restriction enzyme cleavage sites are indicated in italics. [file 1472-6750-11-64-S5.DOC]

| Oligo name | Sequence (5’-3’) | Restriction sites |
| --- | --- | --- |
| Avd_NheI_5’ | ATATT*GCTAGC*TGCACAACCAGCAATGGCAGCCAGAAAGTGCTCGCTGAC | *NheI* |
| Avd_NotI_3’ | TTAATT*GCGGCCGC*CTCCTTCTGTGTGCGCAGGC | *NotI* |
| N118M.1 | GGTCGGCATCATGATCTTCACTC | *-* |
| N118M.2 | GAGTGAAGATCATGATGCCGACC | *-* |
| Avd_AscI_stop_3’ | TTAATT*GGCGCGCC*TCACTCCTTCTGTGTGCGCAG | *AscI* |
| Avd_SfiI_5’ | AATTGC*GGCCCAGCCGGCC*ATGGCCGCCAGAAAGTGCTCGCTGAC | *SfiI* |
| Loop 1-2_R2_5’ | AACATGACCATCGGGGCT | *-* |
| Loop 1-2 _R1_3’ | AGCCCCGATGGTCATGTTNNNNNNNNNNNNNNNGGTCCATTTCCCAGTCAG | *-* |
| 3_4R_2_5’ | TCAAATGAGATCAAAGAG | *-* |
| 3_4R_1_3’ | CTCTTTGATCTCATTTGARNNRNNRNNRNNRNNRNNGATGTAGGTGCCTGTGAATTC | *-* |
